# Supplementary material for: NUAK2 is a critical YAP target in liver cancer
Source: Nat Commun. 2018 Nov 16;9:4834. doi: 10.1038/s41467-018-07394-5 (PMC6240092; doi:10.1038/s41467-018-07394-5)
Supplement: Supplementary file 1 — Supplementary information [file 41467_2018_7394_MOESM1_ESM.pdf]

SUPPLEMENTARY INFORMATION

NUAK2 is a critical YAP target in liver cancer

Yuan et al.

SUPPLEMENTARY FIGURES

Sup. Figure 1.

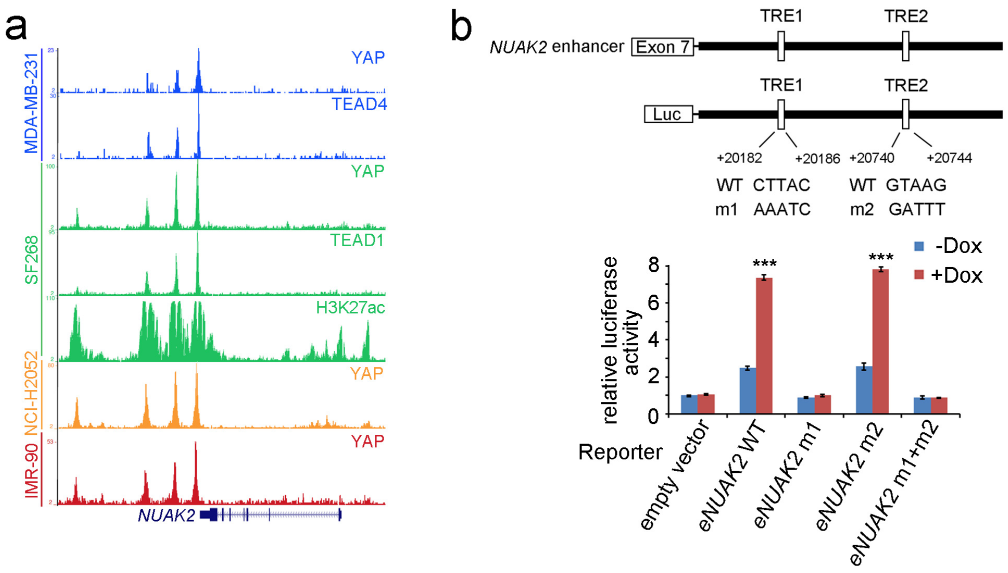

Supplementary Figure 1. YAP binds to *NUAK2* enhancer to regulate its expression.

(a) Genomic tracks display ChIP-seq data for the indicated antibodies around *NUAK2* gene in indicated cell lines. (b) Schematic representations of the regulatory region of *NUAK2* gene and the luciferase reporter constructs. The positions of TEAD response elements (TREs) and the sequences of wild-type (WT) and mutant (m1, m2) TREs are indicated. Enhancer activity assay using Dox-inducible YAP S127A H69 cells transfected with the indicated reporter construct and treated with Dox for 24 hr. Data are mean ± SD; n = 5. The two-tailed, Student's t-test was used to compare between two groups and expressed as P values. *P<0.05, **P<0.01, ***P<0.001.

Sup. Figure 2.

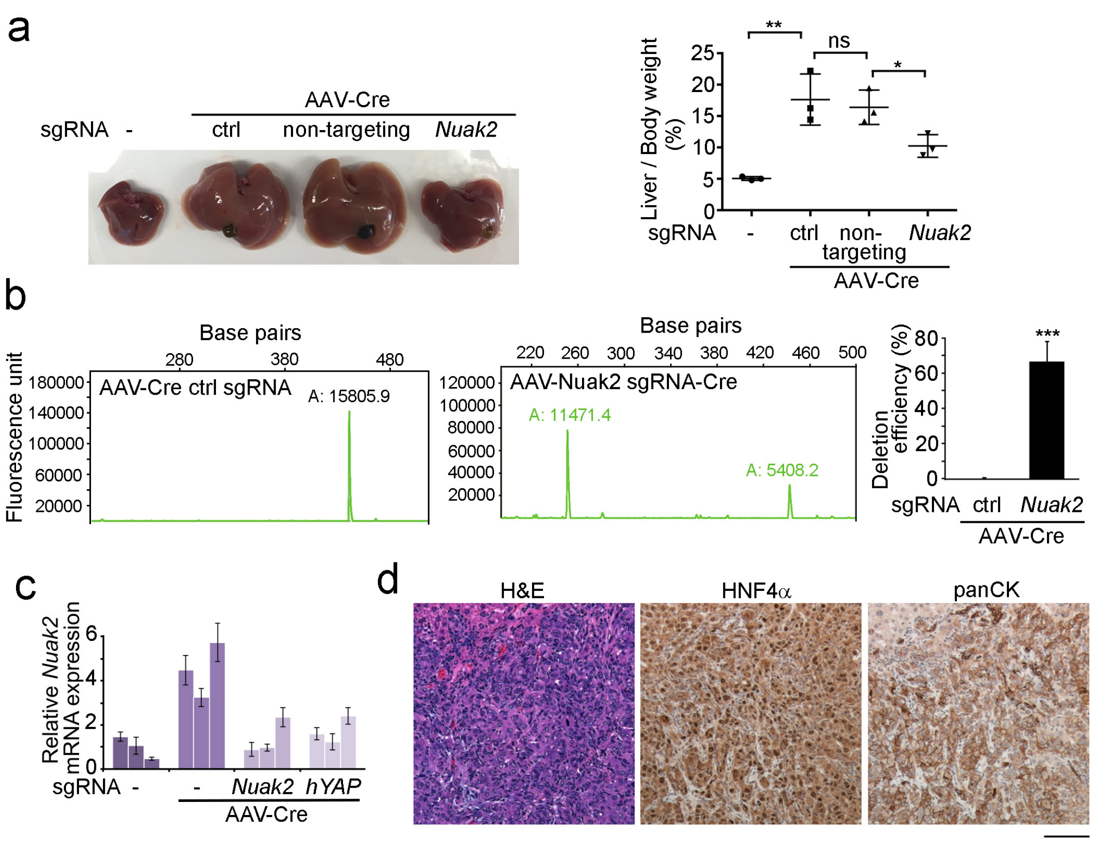

Supplementary Figure 2. The role of Nuak2 knockout in the mouse liver in YAP-driven hepatomegaly and tumorigenesis

(a) Gross morphology of the livers of TetO-YAP:Cas9 transgenic mice infected with indicated AAV virus and placed on Dox for 2 weeks. (Right panel) Liver/Body weight ratio of mice mentioned before was plotted. Data are presented as mean ± SD; n=3. The two-tailed, Student's t-test was used to compare between two groups and expressed as P values. *P<0.05, **P<0.01, ***P<0.001. (b) Representative result of PCR-fragment assay of genomic DNA from TetO-YAP:Cas9 mouse livers infected with high dose ($5X10^{10}$) AAV-Cre or AAV-Nuak2 sgRNA-Cre and gave Dox for 4 days. Since the AAV-Nuak2 sgRNA-Cre contains two sgRNAs targeting *Nuak2*, CRISPR/Cas9 mediated editing would be assumed to generate around 200 bp deletions between two sgRNAs. The short length fragment indicates deletion and the long length fragment indicates wild type. (Right panel) The percentage of deletion

efficiency determined by fragment assay. Data are mean ± SD; n = 3. The two-tailed, Student's t-test was used to compare between two groups and expressed as P values. *P<0.05, **P<0.01, ***P<0.001. (c) qPCR analysis of *Nuak2* expression in TetO-YAP:Cas9 mice livers. Each bar represents a different mouse liver. n = 3 mice, mean ± SD (d) Hematoxylin and eosin (H&E) and IHC analysis of liver tumor sections from TetO-YAP:Cas9 mice infected with control sgRNA. Bar, 50 μm.

Sup. Figure 3.

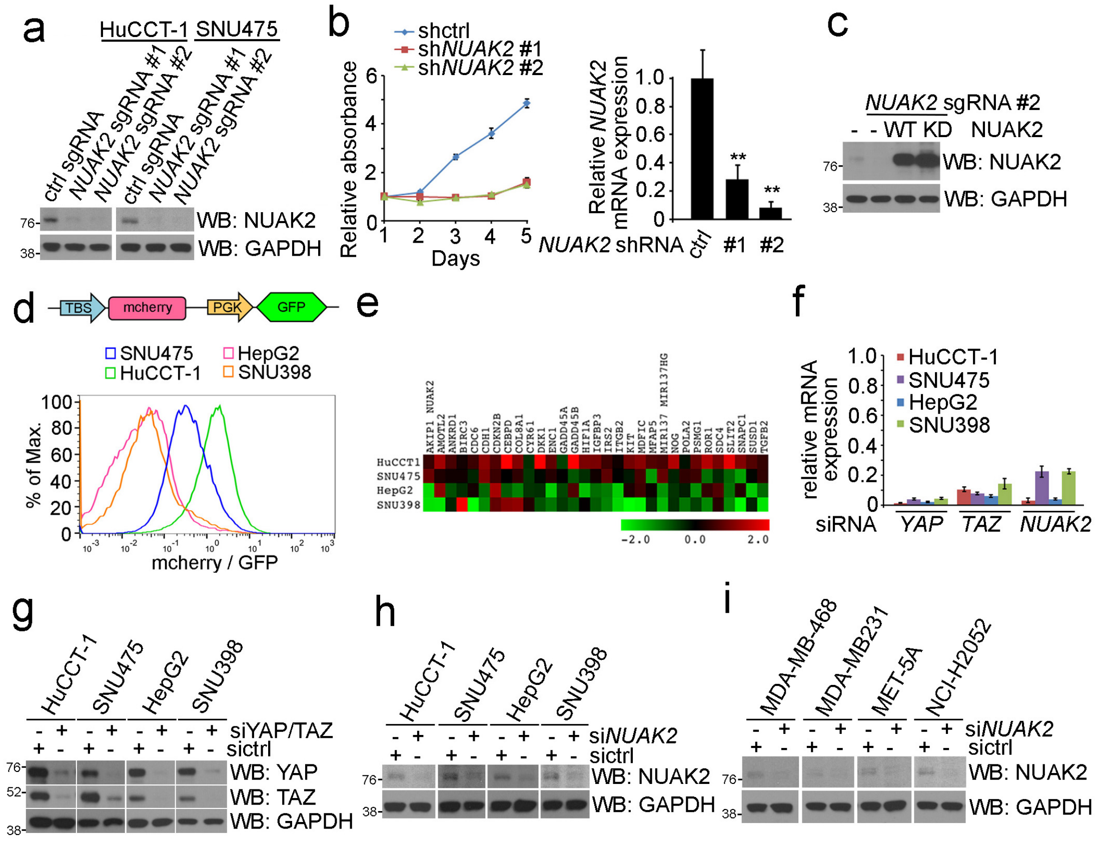

Supplementary Figure 3. NUAK2 is involved in YAP-mediated cell proliferation. Western blot analysis of NUAK2 in HuCCT-1 and SNU475 cells transfected with indicated sgRNAs. (b) Growth curves of HuCCT-1 cells infected with indicated shRNA (left panel) using crystal violet assessment of cell growth. Data are presented as mean ± SD; n=3. qPCR analysis of *NUAK2* expression (right panel). Data are presented as mean ± SD; n=3. The two-tailed, Student's t-test was used to compare between two groups and expressed as P values. *P<0.05, **P<0.01, ***P<0.001. (c) Western blot analysis of NUAK2 in HuCCT-1 cells transfected with scramble or *NUAK2* sgRNA and/or vectors expressing cDNAs encoding wild-type or kinase dead NUAK2. (d) Schematic of lentivirus containing TBS promoter-driven mcherry and PGK promoter driven GFP. The depicted liver cancer cell lines were infected the lentivirus and the cellular mcherry/GFP ratio was measured by FACS. (e) Relative mRNA expression of YAP signature in liver cancer cell lines. (f) qPCR analysis of

YAP, *TAZ* and *NUAK2* expression in liver cancer cell lines. n = 3, mean ± SD. (g) Western blot analysis of YAP and TAZ in indicated cells transfected with indicated siRNAs. (h) Western blot analysis of NUAK2 in indicated cells transfected with scramble or *NUAK2* siRNAs. (i) Western blot analysis of NUAK2 in indicated cells transfected with scramble or *NUAK2* siRNAs

Sup. Figure 4.

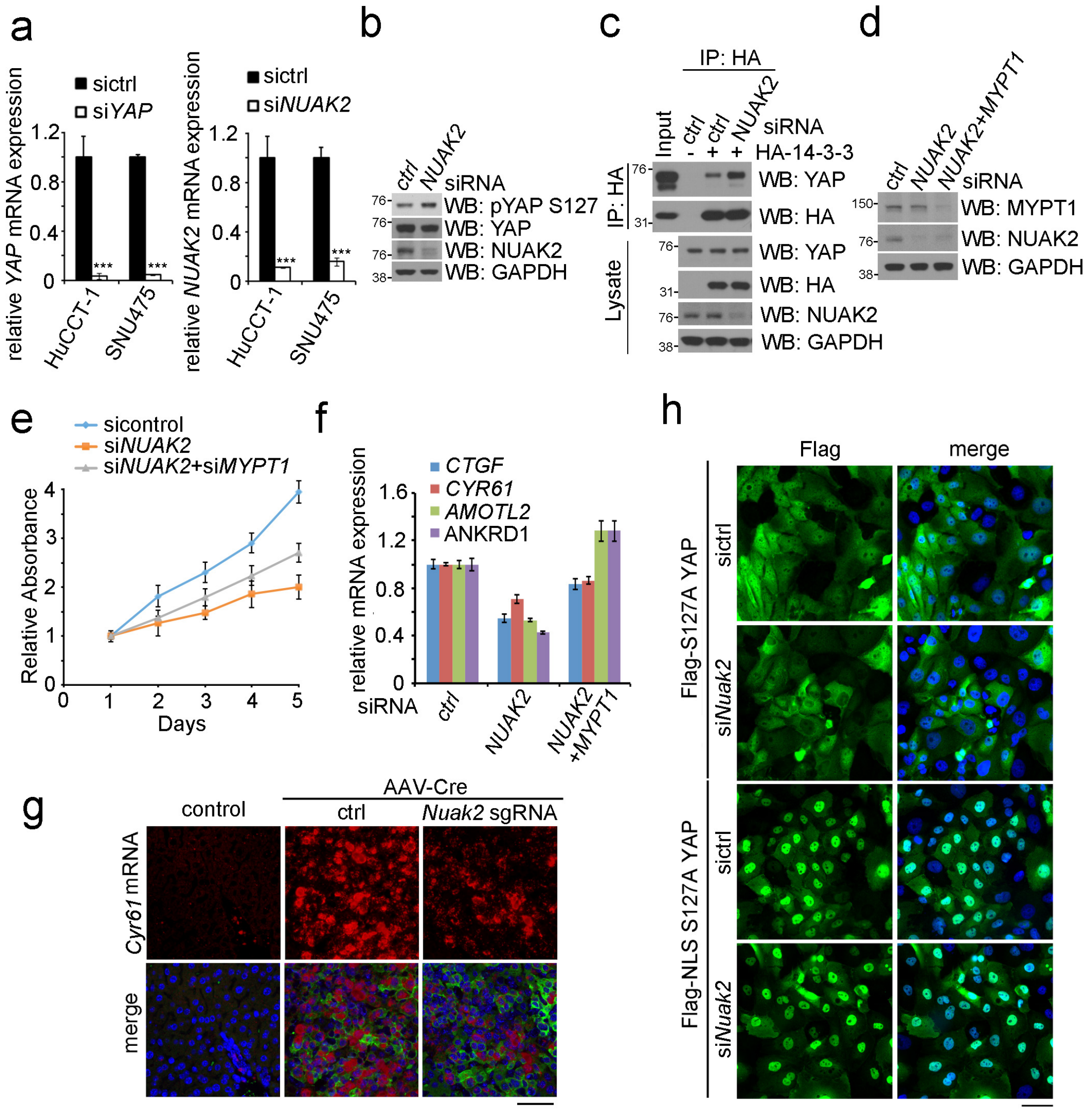

Supplementary Figure 4. The efficiency of NUAK2 or YAP knockdown in HuCCT-1 and SNU475 cells.

(a) qPCR analysis of *YAP* and *NUAK2* expression in HuCCT-1 and SNU475 cells 72 h after siRNA transfection. n = 3, mean ± SD. The two-tailed, Student's t-test was used to compare between two groups and expressed as P values. *P<0.05, **P<0.01, ***P<0.001. (b) Western blot analysis of YAP, phospho-S127 YAP and NUAK2 in HuCCT-1 cells transfected with indicated siRNAs. (c) Co-immunoprecipitation analysis of the interaction between HA-14-3-3 and endogenous YAP in HuCCT -1 cells transfected with the indicated siRNAs and plasmids. (d)

Western blot analysis of NUAK2 and MYPT1 in HuCCT-1 cells transfected with indicated siRNAs. (e) Growth curves of HuCCT-1 cells transfected with indicated siRNA. Data are presented as mean ± SD; n=6. (f) qPCR analysis of YAP downstream genes in HuCCT-1 cells transfected with indicated siRNA. n = 3, mean ± SD. (g) Liver sections were analyzed by RNAscope for *Cyr61* and co-stained with anti-GFP antibody and DAPI. Bar, 50 μm. (h) Immunofluorescent images of HuCCT-1 cells stably expressing Dox-inducible Flag-YAP mutants transfected with indicated siRNAs for 72 hr and stained with Flag antibody and DAPI. Bar, 20 μm.

Sup Figure 5

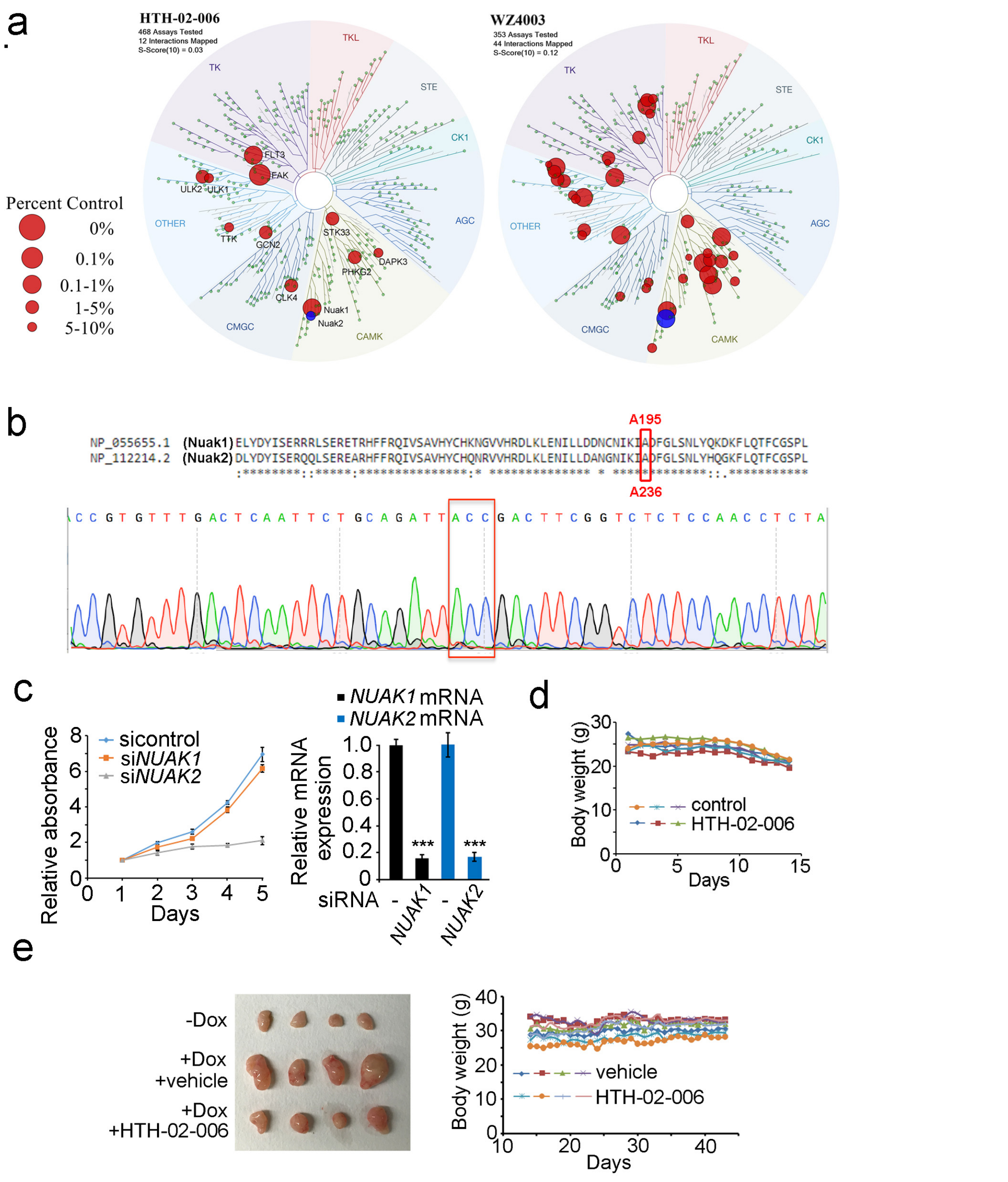

Supplementary Figure 5. HTH-02-006 inhibits cell proliferation and hepatomegaly via regulation of Nuak2.

(a) Biochemical kinase selectivity profiles (KINOMEscan) of HTH-02-006 and WZ4003 were performed against a panel of 468 and 352 kinases, respectively. NUAK2 is labeled in blue. Only kinases with ≤10% retention are displayed. The S-

Score(10) indicates the percentage of kinases within the panel that demonstrate <10% retention on the solid support after inhibitor treatment. (b) Alignment of NUAK1 and NUAK2 showing the conservation of the residue that mediated the binding with WZ4003/HTH-02-006. Genomic DNA around Nuak2 amino acid 236 was PCR amplified and sequenced. Sequence at 236 residue was confirmed to be mutated from Alanine to Threonine. (c) Cell growth curves of HuCCT-1 cells transfected with either scramble, NUAK1 or NUAK2 siRNA using crystal violet assessment of cell growth (left panel). Data are presented as mean ± SD. n=5. Right, Quantitative real-time PCR analysis of NUAK1 and NUAK2 expression levels. Data are presented as mean ± SD. n=3. The two-tailed, Student's t-test was used to compare between two groups and expressed as P values. *P<0.05, **P<0.01, ***P<0.001. (d) Body weight of TetO-YAP S127A mice treated with vehicle control or HTH-02-006 (10 mg/kg, twice daily, i.p.) over the duration of drug treatment. (e) Gross morphology of the tumors of xenograft model (Left panel). Body weight of Nude mice treated with vehicle control or HTH-02-006 (10 mg/kg, twice daily, i.p.) over the duration of drug treatment (Right panel).

Sup Figure 6

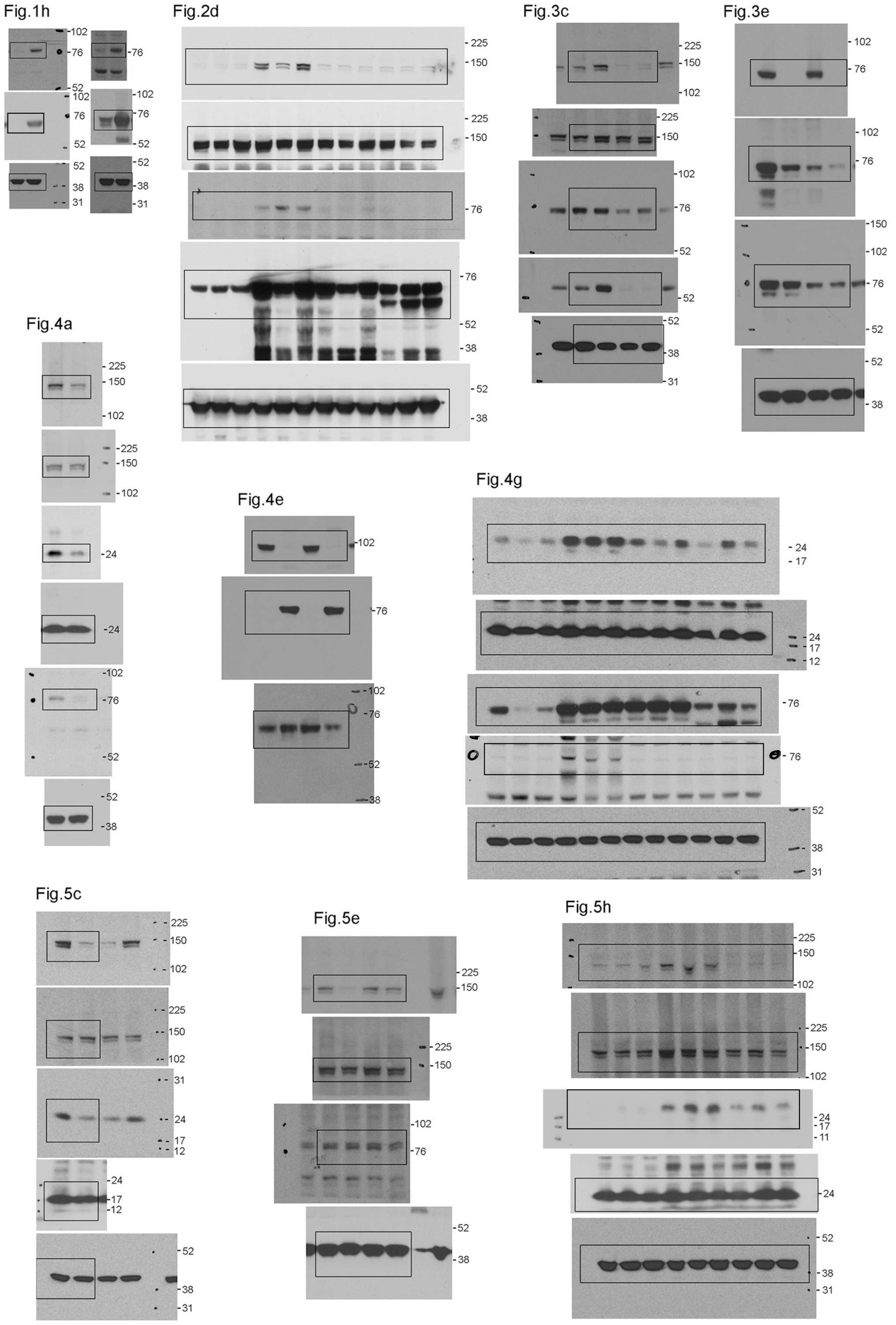

Supplementary Figure 6. Uncropped images of Western blots experiments of Figure 1 50 Figure 5.

Molecular weight markers are indicated. Rectanges represent the cropped images shown in Figures.

Supplementary Table 1. 14 YAP transcriptional targets by integration of ChIP-seq and gene expressing profile.

Mdfic	*Bicc1*	*Fstl1*	*Ankrd1*
Cdc6	*Wtip*	*Tgfb2*	*Amotl2*
Veph1	*Kif18b*	*Nuak2*	*Cyr61*
Rttn	*Gadd45b*		

Supplementary Table 2.

Primer name	Forward primer (5’-3’)	Reverse primer (5’-3’)	Application
hNUAK2	GATGCACATACGGAGGGAGATT	ATCACGATCTTGCTGCTGTTCT	qPCR
hRPS18	TTCGGAACTGAGGCCATGAT	TTTCGCTCTGGTCCGTCTTG	qPCR
hCTGF	GAAGCTGACCTGGAAGAGAACA	CGTCGGTACATACTCCACAGAA	qPCR
hCYR61	CATTCCTCTGTGTCCCCAAGAA	TACTATCCTCGTCACAGACCCA	qPCR
hNUAK1	ATATACTGCTCGATGACAACTGC	CATAGAGTGGACTCCCACAAAAC	qPCR
mNuak2	CAGGCATTTCTTCCGACAGATC	GAGAGGCCAAAGTCAGCAAT	qPCR
mRRN18s	TTGACGGAAGGGCACCACCAG	GCACCACCACCCACGGAATCG	qPCR

Supplementary Method 1.

Synthetic scheme for Compound HTH-02-006

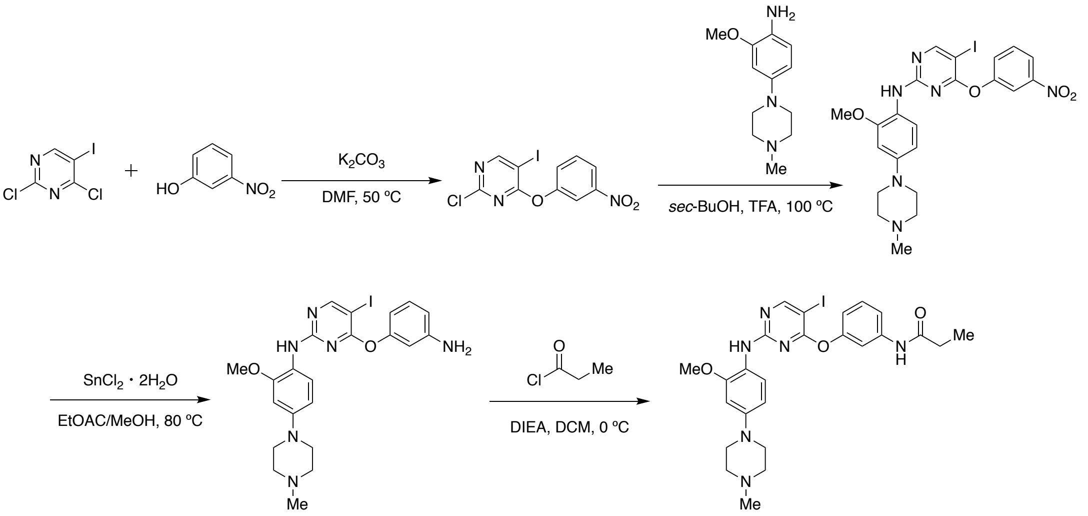

Synthesis of Intermediates

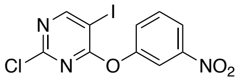

2-chloro-5-iodo-4-(3-nitrophenoxy)pyrimidine

To a solution of 3-nitrophenol (304 mg, 2.183 mmol, 1 eq) in DMF (5 mL) was added potassium carbonate (603 mg, 4.366 mmol, 2 eq) and 2,4-dichloro-5-iodopyrimidine (600 mg, 2.183 mmol, 1 eq). The reaction was heated to 50 ºC for 2 hours. The reaction mixture was filtered. The filtrate was dilute with ethyl acetate and washed with water three times. The organic layer was dried over anhydrous magnesium sulfate and concentrated to afford 700 mg (84.9%) of solid, which was used without further purification.

MS m/z: 377.91 (M+1)

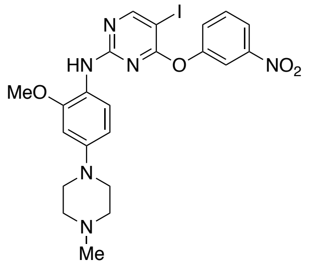

<u>5-iodo-*N*-(2-methoxy-4-(4-methylpiperazin-1-yl)phenyl)-4-(3-nitrophenoxy)pyrimidin-2-amine</u>

To a solution of 2-chloro-5-iodo-4-(3-nitrophenoxy)pyrimidine (700 mg, 1.85 mmol, 1 eq) in *sec*-BuOH (8 mL) was added 2-methoxy-4-(4-methylpiperazin-1-yl)benzenamine (533 mg, 2.41 mmol, 1.3 eq) and TFA (2 mL). The slurry was heated to 100 ºC for 3 hours. The reaction mixture was allowed to cool to room temperature, and was neutralized with a saturated aqueous sodium bicarbonate solution. The aqueous mixture was then extracted with chloroform/isopropanol (4:1) three times. The organic layer was dried over anhydrous magnesium sulfate and concentrated. The crude product was purified using flash chromatography with dichloromethane and 1.75 N ammonia in methanol to afford 700 mg (67.3%) of solid.

MS m/z: 563.08 (M+1)

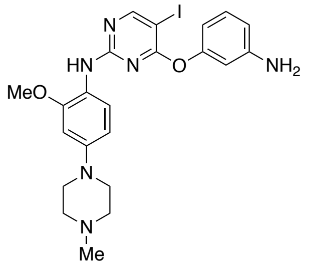

<u>4-(3-aminophenoxy)-5-iodo-*N*-(2-methoxy-4-(4-methylpiperazin-1-yl)phenyl)pyrimidin-2-amine</u>

To 5-iodo-*N*-(2-methoxy-4-(4-methylpiperazin-1-yl)phenyl)-4-(3-nitrophenoxy)pyrimidin-2-amine (810 mg, 1.44 mmol, 1 eq) in ethyl acetate/methanol (1:1, 14 mL) was added tin chloride dihydrate (1.3 g, 5.76 mmol, 4 eq). The reaction was heated to 80 ºC for 3 hours. The reaction mixture was cooled to room

temperature, and was extracted with saturated aqueous sodium bicarbonate solution and chloroform/isopropanol (4:1) three times. The organic layer was dried over anhydrous magnesium sulfate and concentrated. The crude product was purified using flash chromatography with dichloromethane and 1.75 N ammonia in methanol to afford 355 mg (46.3%) of solid.

MS m/z: 533.11 (M+1)

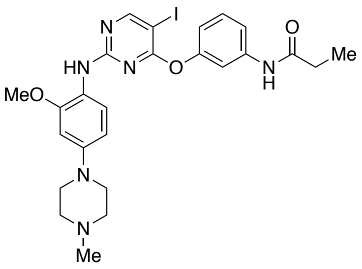

<u>*N*-(3-((5-iodo-2-((2-methoxy-4-(4-methylpiperazin-1-yl)phenyl)amino)pyrimidin-4-yl)oxy)phenyl)propionamide (HTH-02-006)</u>

To a solution of 4-(3-aminophenoxy)-5-iodo-*N*-(2-methoxy-4-(4-methylpiperazin-1-yl)phenyl)pyrimidin-2-amine (355 mg, 0.667 mmol, 1 eq) and diisopropyethylamine (129.3 mg, 1 mmol, 1.5 eq) in dichloromethane (1.5 mL) at 0 ºC was added propionyl chloride (68 mg, 0.734 mmol, 1.1 eq) drop-wise. The reaction was stirred for 1 hour. 248 mg (63.2 %) of the titled compound was obtained as solid after recrystallization using ethyl acetate and petroleum ether without further purification.

MS m/z: 589.13 (M+1).

^{1}H NMR (500 MHz, DMSO-d_6) δ 10.02 (s, 1H), 8.47 (s, 1H), 8.01 (s, 1H), 7.51 (t, *J* = 2.1 Hz, 1H), 7.46 (d, *J* = 8.2 Hz, 1H), 7.36 (t, *J* = 8.2 Hz, 1H), 7.23 (d, *J* = 8.2 Hz, 1H), 6.87 (ddd, *J* = 8.0, 2.3, 0.8 Hz, 1H), 6.52 (d, *J* = 2.3 Hz, 1H), 6.21-6.13 (br), 3.73 (s, 3H), 3.05 (t, *J* = 4.9 Hz, 4H), 2.43 (t, *J* = 4.9 Hz, 4H), 2.32 (q, *J* = 7.6 Hz, 2H), 2.21 (s, 3H), 1.07 (t, *J* = 7.6 Hz, 3H).
